# Study of Health and Activity in Preschool Environments (SHAPES): Study protocol for a randomized trial evaluating a multi-component physical activity intervention in preschool children

**DOI:** 10.1186/1471-2458-13-728

**Published:** 2013-08-07

**Authors:** Karin A Pfeiffer, Ruth P Saunders, William H Brown, Marsha Dowda, Cheryl L Addy, Russell R Pate

**Affiliations:** 1 Department of Kinesiology, Michigan State University, 308 West Circle Drive, 27R Intramural Rec Sports- Circle, East Lansing, MI 48824, USA; 2 Department of Heath Education and Behavior, University of South Carolina, 800 Sumter Street, Columbia, SC 29208, USA; 3 Department of Educational Studies, University of South Carolina, 801 Sumter Street, Columbia, SC 29208, USA; 4 Department of Epidemiology and Biostatistics, University of South Carolina, 800 Sumter Street, Columbia, SC 29208, USA; 5 Children’s Physical Activity Research Group Department of Exercise Science, University of South Carolina, 921 Assembly Street, Columbia, SC 29208, USA

**Keywords:** Young children, Policy, Pre-K, School, Childcare

## Abstract

**Background:**

Physical inactivity is a recognized public health concern. Inadequate proportions of children in the U.S, including those of preschool age, are meeting physical activity recommendations. In response to low numbers of preschool children attaining appropriate physical activity levels, combined with the large number of young children who attend preschool, researchers have identified the need to devise interventions to increase physical activity at preschools. However, few multi-component interventions to increase physical activity in preschool children exist. The aims of this study were to observe the effects of a multi-component intervention on physical activity, sedentary behavior, and physical activity energy expenditure in 3-5 year-old children; identify factors that associate with change in those variables; and evaluate the process of implementing the multi-component intervention. The purpose of this manuscript is to describe the study design and intervention protocol.

**Methods/design:**

The overall design of the Study of Health and Activity in Preschool Environments (SHAPES) was a two-year randomized trial (nested cohort design), with two conditions, two measurement occasions, and preschool serving as the unit of analysis. Sixteen schools (eight intervention and eight control) were enrolled. The intervention protocol was based on the social ecological model and included four main components: (a) indoor physical activity (“move inside”), (b) recess (“move outside”), (c) daily lessons (“move to learn”), and (d) social environment. Components were implemented using teacher and administrator trainings and workshops, site support visits, newsletters, and self-monitoring methods. Outcomes included accelerometer assessment of physical activity, sedentary behavior, and physical activity energy expenditure; weight status; and demographic factors; family/home social and physical environment; and parental characteristics. An extensive process evaluation battery was also used to monitor dose delivered by interventionists, completeness of intervention component delivery by teachers, and fidelity of teachers’ implementation.

**Discussion:**

The study will address important gaps relative to increasing physical activity in preschool children. Few studies to date have incorporated a multi-component approach, rigorous measurement protocol, and thorough evaluation of intervention implementation.

**Trial registration:**

NCT01885325

## Background

Physical activity during young childhood is related to favorable levels of positive health outcomes such as cardiovascular disease risk factors (e.g., blood lipids, blood pressure) [1], body composition [2], motor skill development [3], and psychosocial characteristics [4]. Current U.S. recommendations are for preschool children to obtain at least 15 minutes per hour of at least light intensity physical activity while they are in childcare [5]. Inadequate proportions of preschool children in the U.S. [6], Australia [7], and other countries [8] are meeting either the prior or current recommendations. Thus, several researchers have noted the importance of interventions to increase physical activity during the preschool years [9,10], specifically citing preschools as important settings [9].

Over half of young children (3-5 year olds not in kindergarten) in the U.S. spend time in center-based preschool settings [11]. Researchers have shown that preschools are related to young children’s physical activity [12-14], indicating the importance for addressing physical inactivity in those settings. Preschools are complex settings because children spend time in different locations (indoors versus outdoors) and different contexts within those locations, such as circle/group time or pre-academic learning time when indoors versus open space or fixed equipment when outdoors. Specific preschool characteristics that have been associated with children’s physical activity include program quality [15], environmental variables (e.g., portable equipment, larger playgrounds) [15], and social variables (e.g., teacher arranged activities while indoors, child-initiated activities while outdoors) [16]. Effective physical activity interventions in preschools need to account for these variations in setting characteristics.

Although researchers have identified physical activity-promoting characteristics in preschools, there have been relatively few preschool physical activity intervention studies thus far [9,17], particularly randomized controlled trials [9,10]. Existing randomized controlled trials have shown ability to increase physical activity within the preschool setting [18-22]; however, these studies were conducted in different countries or specific cultural groups, were relatively short in duration, or were implemented primarily by well-trained interventionists and not children’s teachers. To address the low prevalence of meeting physical activity recommendations and lack of evidence regarding physical activity interventions in preschool children in the U.S., we designed the Study of Health and Activity in Preschool Environments (SHAPES) using known activity-promoting characteristics. SHAPES is a randomized trial designed to increase physical activity within preschool settings.

The aims of SHAPES were to (a) observe the effects of a multi-component intervention on physical activity, sedentary behavior, and physical activity energy expenditure in 3-5 year-old children; (b) identify factors that associate with change in physical activity, sedentary behavior, and physical activity energy expenditure during a school year in preschool children; and (c) evaluate the process of implementing a multi-component preschool intervention to increase physical activity and reduce sedentary behavior in 3-5 year-old children. SHAPES interventionists employed a flexible and adaptive approach to incorporating physical activity opportunities throughout the entire preschool day. Interventionists worked with teachers (e.g., workshops, on-site consultations) to develop and adapt their own daily activities to their specific classrooms. The goal was to maximize physical activity opportunities throughout the preschool day including outdoor play and classroom activities (e.g., center time, large group activities, pre-academic lessons). The purpose of this manuscript is to describe the SHAPES study design and intervention protocol.

## Methods

### Design

The overall design of SHAPES was a two-year randomized trial (nested cohort design), with two conditions, two measurement occasions, and preschool serving as the unit of analysis (see Table 1 for logic model for the study). Sixteen schools (eight intervention and eight control) were invited to participate and met the following eligibility criteria: the program was educative in nature and met for at least three hours per day and for 180 days per school year; the curriculum met state standards and focused on developmental and pre-academic skills for preschoolers (e.g., emergent literacy, mathematics, social development). The two major types of schools involved were public and private four-year-old pre-kindergartens and tuition-based programs, most of which were operated by religious or commercial organizations. A stratified random sample of schools meeting the described criteria was drawn and invited to participate in the study. If a preschool declined to participate, a replacement program was selected from the same stratum. Baseline and follow-up data were collected for each school year of the study. The duration of the first intervention year was approximately 15-25 weeks, and the second intervention year was approximately 31 weeks in duration. During the first year of the study, a particular group of students was assessed at baseline and follow-up. During the second year of the study, another cohort (i.e., new students in a particular teacher’s classroom) was assessed at baseline and follow-up; thus, the duration of the intervention at a given preschool was two years, but two separate cohorts were assessed in the measurement protocol (one each year). The study was approved by the University of South Carolina’s Institutional Review Board.

**Table 1:** SHAPES logic model

** Inputs**		** Outputs**		** Outcomes**
**What we invest and**	**Who is reached and**	**Changes made by “change**	**Organizational changes**	**Individual behavior**
**what we do**	**expected effects**	**agents” (intervention**	**in preschool setting**	**change (activity-related**
		**implementation)**		**behaviors)**
SHAPES intervention staff provide training, ongoing assistance, and small amounts of funding (for small equipment and supplies) to…	Preschool teachers and assistant teachers in 4K classrooms who will carry out the SHAPES intervention by…	Providing physical education/indoor physical activity for at least 10 minutes per day (“move inside”); recess for two sessions of at least 20 minutes per day, with at least one five-minute structured opportunity (“move outside”); daily lessons with at least two, five-minute sessions per day (“move to learn”); and a supportive social environment, which will result in….	An improved preschool instructional and social environment that promotes and supports increased PA and decreased sedentary behavior in children, which will result in…	Increased moderate-to-vigorous physical activity and energy expenditure, and reduced sedentary time in preschool-aged children and
				Maintenance or improvement of body mass index

### Participants

Participants were children who were assigned to the 35 4-year-old classrooms in preschools that agreed to participate in the study. Approximately 13-37 children, from one to seven classrooms per preschool, were recruited and met eligibility criteria during each school year. The total sample across two years was approximately 500 children. To be eligible for the study, children’s parents must have provided informed consent and indicated that they expected their child to remain enrolled in the same preschool through the end of the current academic year. All parents of children assigned to 4-year-old classrooms in participating preschools were invited to involve their children in the study. Children were excluded from the study if they had a disability that would invalidate accelerometry as a measure of physical activity (e.g., use of wheelchair, employment of a walker) or if they were outside the 3-5 year-old age range. If there were siblings in the sample, data from only one child per family will be used for analyses.

### Sample size

For the first study aim, sample size was calculated based on Murray’s formula for calculating detectable difference of a mixed-model ANCOVA [23]. Variance components for preschool physical activity were estimated from the research team’s prior work [14]. Detectable differences for all measures were between 0.30 and 0.43 standard deviation units (small to medium sized effects), which translated to detectable differences of 0.62 minutes of MVPA per hour, 1.63 minutes of sedentary time per hour, and 0.33 ml/kg/min of energy expenditure, assuming a conservative estimate of measuring 25 children per preschool. For the second aim, calculations were based on a mixed-model regression approach, with condition (intervention versus control) as a fixed effect and preschool nested within condition as a random effect. Because more degrees of freedom were accounted for in this analysis, the calculations for the first aim estimated a more conservative sample size than what was necessary for the second aim. Power calculations were not needed for the third aim, as this type of evaluation is predominantly descriptive in nature.

### Intervention description

#### Theoretical framework

The intervention was based on a social ecological model of health behavior [24]. Within the social ecological model, behavior is influenced at multiple levels. In preschool settings, this includes individual, instructional, social and physical variables. Factors at all of these levels have the ability to affect physical activity levels of preschoolers. Our team hypothesized that increasing physical activity promoting practices and policies (i.e., instructional and environmental factors) in the preschool classrooms would increase the physical activity levels of preschoolers in these settings. The classroom level changes included providing activity resources, influencing teaching practices, and promoting adjustment of the daily schedule to include additional opportunities for children’s physical activity. Individual level factors will be taken into account as well, during statistical analyses.

#### Intervention protocol

The SHAPES intervention was considered a partnership between intervention team members and preschool teachers and was designed to be flexible and adaptive to preschool settings. The SHAPES intervention was based on four major components: (a) physical education or indoor physical activity for at least 10 minutes per day (“move inside”), (b) recess for two sessions of at least 20 minutes per day, with at least one five-minute structured physical activity opportunity (“move outside”), (c) daily classroom lessons with at least two, five-minute sessions per day (“move to learn”), and (d) enhanced social support for physical activity. The intervention staff provided “essential elements” to be implemented (see Table 2), but preschool teachers were encouraged to adapt any intervention components to improve their classrooms with respect to children’s physical activity. Thus, SHAPES represented various methods for incorporating physical activity into the preschool day and was not a prescribed curriculum. Central elements of implementation were teachers’ trainings and workshops, site support visits, newsletters, and self-monitoring activities. Interventionists provided materials, supplies, and examples for all intervention components, but teachers were encouraged to modify individual activities, with interventionists’ assistance, to meet their children’s needs and capabilities. Original materials consisted of information adapted from existing curricula such as Animal Trackers [25], but as the intervention progressed, the intervention staff focused on activities created by teachers and provided the activities and accompanying materials to the other teachers in the intervention settings. Teachers were always recognized individually and publically (e.g., at workshops, in newsletters) for their contributions. The four major intervention components are further described in the following text.

**Table 2:** Essential Elements of the Study of Health and Activity in Preschool Environments (SHAPES) Intervention

**Component**	**Delivery plan**
Physical Education or Indoor Physical Activity “Move Inside”	1. Provided at least 10 minutes per day
	2. Children active for 50% of Move Inside time
	3. Children enjoy physical activity (PA)
Recess “Move Outside”	4. At least two 20-minute sessions of recess provided daily
	5. Teachers provide at least 5 minutes of structured activity daily
	6. Children enjoy PA
Daily Lessons “Move to Learn”	7. PA is integrated into pre-academic lessons for at least two 5-minute sessions
	8. Children enjoy PA in lessons
Social Environment	9. Teachers actively participate in PA with children during “Move Inside”, “Move Outside”, “Move to Learn”, and any other PA time
	10. Teachers verbally encourage PA in children during “Move Inside”, “Move Outside”, “Move to Learn”, and any other PA time

### Move inside

Formal physical education in preschools is typically either non-existent or not consistently offered. The idea behind “Move Inside” was to encourage teachers to provide opportunities each day for children to engage in moderate-to-vigorous physical activity (MVPA). The goal set for teachers was to provide at least 10 minutes daily of indoor MVPA that was not part of recess or academic lessons. This included activities such as theme-based obstacle courses, dancing to music provided to teachers on CDs, or activities that focused on development of particular motor skills (e.g., hopscotch-type activities).

### Move outside

Previous work from our investigative team showed children to be more physically active when they are outdoors [16]. Thus, teachers were asked to provide outdoor recess whenever possible. If the weather was not conducive to being outdoors, teachers were encouraged to provide indoor recess opportunities of equal duration and intensity. The goal was for teachers to provide at least two, 20-minute recesses daily. As part of the 20-minute “Move Outside” activities, teachers were asked to lead at least one, 5-minute structured activity opportunity per day. Structured recess activities included games like tag, Track Team (where children jog around the playground with teachers and peers), or Buzzing Bees (where children “buzz” around while flapping their elbows and searching for nectar- either inside or outside).

### Move to learn

Even at the preschool level, teachers and preschool directors are concerned with delivering appropriate levels of academic content. The “Move to Learn” intervention component incorporated physical activity into pre-academic lessons to support children’s learning (e.g., recitations while moving, counting motor movements). Teachers were encouraged to be creative with how they integrated physical activity into daily schedules and were asked to conduct two, 5-minute physically active lessons per day for a total of 10 minutes daily. Examples were singing the alphabet while being physically active (e.g., F stands for frog; now jump, jump, jump on a lily pad), acting out stories (e.g., “Going on a Bear Hunt”), or counting with large muscle motor movements (e.g., jumping jacks).

### Social environment

SHAPES personnel addressed the social environment in two ways. First, they asked teachers to participate in physical activities along with the preschool children whenever possible. Second, they recommended that teachers verbally encourage preschoolers’ participation during planned and unplanned physical activities. The verbal encouragement included acknowledging physical activity behaviors (e.g., “You are doing a great job kicking and chasing the ball!”) and promoting additional physical activities (e.g., “Can you show me how to run like a lioness?”). Additionally, we asked teachers not to discourage safe physical activity at appropriate times or use physical activity as a punishment for children’s problem behaviors.

#### Intervention implementation approach

##### Trainings and workshops

Each preschool program received individual initial training on-site during the first year of the study. Initial trainings lasted two-to-three hours and were scheduled at convenient times for teachers. Components during initial trainings included background information concerning the need to enhance young children’s physical activity, explanations of intervention components with examples, and discussions regarding potential barriers to intervention implementation. The interventionists also distributed physical activity supplies at this time. Throughout the remainder of the intervention, interventionists offered five workshops, which were held at convenient times for teachers. At each workshop, the SHAPES personnel provided food, additional physical activity equipment, a gift card incentive, and childcare for teachers’ children. The goals of the workshops were to provide teachers with the knowledge and skills necessary for implementation and adaptations of the intervention components. We designed the workshops in a partnership format so that teachers could interact and learn from both interventionists and each other. The intervention personnel chose workshop topics based on their observations of teachers in the field and teachers’ requests for information related to children’s physical activity. Attendance was not mandatory. Across both years of the intervention, if there was teacher turnover, the interventionists individually trained the new teachers as soon as possible and invited them to participate in any remaining workshops.

##### Site support visits

Throughout both years of the intervention, one-to-three interventionists made site visits to participating preschool classrooms. Visits during the first year focused on interventionists arranging and leading physical activities to provide specific examples for the teachers. Additionally, interventionists addressed teachers’ concerns. Visits during the second year were more frequent, with two visits per month taking place. One of the visits consisted of the interventionists actively participating in teacher-led classroom activities. The other took place during a quiet time, usually nap, where interventionists and teachers could discuss, plan and problem solve with issues regarding enhancing children’s physical activity.

##### Newsletters

All intervention classrooms received electronically delivered and hand-delivered newsletters, and parents of participating children received hard copy newsletters. Interventionists created separate newsletters for parents and teachers. The teachers’ newsletters included tips, ideas, and physical activity suggestions from interventionists and other teachers. The parents’ newsletters contained background information explaining the intervention components and ideas for fun family physical activities. The newsletters highlighted and described activities performed by teachers, along with tips from the teachers to improve children’s physical activity. During the first year of the study, intervention personnel created a password-protected website as a resource for a sharing and networking community, but they discontinued use of the website in the second year due to infrequent teacher participation.

##### Self-assessment methods

Interventionists provided a “SHAPES Jar” to each classroom teacher during the intervention. Teachers were asked to add blocks to the jar to track participation in intervention components. The jar was a physical reminder and general indicator of the classroom activities that did not involve excessive paperwork, and could involve children, who often helped and talked about physical activities while they filled the jar during circle time or other large group activities.

#### Measurement protocol

##### Accelerometry

Physical activity, sedentary behavior, and physical activity energy expenditure were measured using the ActiGraph accelerometer (ActiGraph models GT1M and GT3X; Pensacola, FL). The ActiGraph is a uniaxial (GT1M) or triaxial (GT3X) accelerometer that measures acceleration in the vertical plane (GT1M) or all three planes (GT3X); because multiple models were used in the present study, only data from the vertical plane were analyzed. Accelerometers capture and filter acceleration signals that are digitized and recorded as count values that are stored in user-defined intervals. For the present study, the monitors were initialized to save data in 15-second intervals (epochs) to detect the spontaneous physical activity of three- to five-year-old children.

Participants were instructed to wear the accelerometers on an elastic belt on the right hip (anterior to the iliac crest) during all waking hours, including naps at school, for one week (weekdays only). Parents received instruction sheets to assist their children in complying with procedures. For analyses, up to five days of weekday data were used, and school day physical activity were analyzed separately from total day physical activity. In order to be included in the school day analyses, participants must have worn the monitor for 50% of the preschool day. In addition to the preschool day requirement, participants must have worn the monitor for four hours while not in school to be included in the total day analysis. Additionally, weekdays on which the children did not attend preschool were not included in the analyses, because those days did not represent typical weekdays. Periods of 60 minutes or more of continuous zeroes were considered non-wear times and not included in the calculation of total wear time. Participants with fewer than three days of monitor wear were excluded from the analyses.

Cutpoints and an energy expenditure equation developed specifically for the preschool age-group were used to categorize each minute of wear as sedentary (<200 counts/min), light (200-1679 counts/min), moderate-to-vigorous (≥ 1680 counts/min) or vigorous physical activity (≥ 3368 counts/min) [26]. Minutes per hour of moderate-to-vigorous (MVPA) and sedentary activity were calculated for the total group and for each gender separately. Additionally, energy expenditure was calculated from the equation VO_2_ = 10.0714 + (0.02366*count/15-sec) [26]. A VO_2_ value was calculated from each 15-second count, and values were averaged over the time the monitor was worn. Resting VO_2_ was calculated using the Schofield equation [27], which includes sex, weight (kg) and height (m). To convert VO_2_ to physical activity energy expenditure, values were converted to kcal (VO_2_/1000 X weight)*5), with resting energy expenditure (MJ/24h *238.85)/1440) subtracted. Data were expressed relative to body weight in kcal/kg/min.

##### Anthropometric measures

Participants’ heights were measured to the nearest 0.1 cm using a portable stadiometer (Shorr Productions; Olney, MD). Weights were measured to the nearest 0.1 kg using an electronic scale (Seca, Model 770; Hamburg, Germany). The average of two measurements was used for both height and weight. Body Mass Index (BMI) was calculated and expressed as kg/m^2^. For statistical analyses, BMI Z scores were created by assessing the deviation of each participant’s value from the mean values reported in the CDC growth charts (http://www.cdc.gov/growthcharts). Waist circumference was measured twice, at the level of the umbilicus, to the nearest 0.1 cm using a measurement tape.

##### Parent survey

One parent or guardian for each child completed a survey to assess demographic, family/home social and physical environment, and parental characteristics that were considered potential correlates of children’s physical activity and were consistent with the social ecological framework for the study. The survey was adapted from Sallis and colleagues [28,29]. Adults reported their children’s dates of birth, genders, and ethnicities (African American, White, Other) as well as their own relationships to participating children. Additionally, we asked for respondents’ ages (e.g., under 25, 25-34, 35-44), marital status, educational attainments as an indicator of socioeconomic status, heights, weights, and ethnic backgrounds. Adults’ BMI was calculated from parent self-reported heights and weights and expressed as kg/m^2^. Adults’ participation in physical activity was assessed with the Baecke questionnaire [30]. Family support for physical activity was calculated as the average of responses to five items each regarding frequency of (a) encouragement of physical activity for the participating children, (b) participation in physical activity with the children, (c) provision of transportation to physical activity facilities, (d) watching the children during physical activities, and (e) telling children that physical activity is good for them, responding adults and another household adults (when applicable) [28]. The responding parents also indicated the number of the participating children’s siblings living at home.

Home equipment for physical activity was assessed using a checklist of items found in homes and outdoor play areas. Responding adults reported the items that were used by the participating children and a sum of items was calculated for each child. Park distances, park usages, and park safety were assessed by asking adults to indicate the distance from their homes to the nearest park where their children could be physically active or play sports, how often the adults took their children to that park, and the parents’ perceptions of the reputation of the closest park [28]. Adults also reported their perceptions of their children’s athletic competence compared to other children of the same age and sex, number of sports teams and organized physical activity programs in which the children participated, and amount of screen time [28].

##### Data analysis

Descriptive characteristics were calculated for demographic variables and physical activity by wave and sex. For the first study aim, three mixed-model ANCOVAs, following intention-to-treat principles [23], were used to examine the effects of the SHAPES intervention on total physical activity (min/hr), moderate-to-vigorous physical activity (min/hr), sedentary behavior (min/hr), and physical activity energy expenditure (kcal/kg/min). The first model adjusted for the baseline physical activity variable and wave. The second model added demographic variables (i.e., gender, race and parent education). The third model adjusted for whether the school day was half-day (3-4 hours) or total day (6.5 to 7 hours). We used multiple imputation (data augmentation using Markov Chain Monte Carlo generation) to replace missing outcome data for the physical activity variables, height, and weight for the follow-up measure. For the second aim, a mixed-model ANCOVA was used, testing the interaction of group by factor. In all analyses, preschool was treated as a random variable with students nested in school and group (intervention versus control). Eight children with a twin or sibling included in the study were excluded from the analyses.

### Process evaluation

Investigators systematically monitored “dose” delivered by interventionists (i.e., SHAPES trainings, site visits, resources, materials, and other supports), completeness of intervention component delivery by teachers (i.e., teachers’ providing physical activity opportunities through “Move In”, “Move Out”, and “Move to Learn”), and fidelity of teachers’ implementation of SHAPES (i.e., children are active and enjoying physical activity opportunities and teachers are encouraging and participating in physical activity with children). We also assessed preschool practices related to physical activity, teachers’ satisfaction with and dispositions toward training and support activities and intervention components, contextual elements (e.g., classroom physical environments, teacher turnover), and preschool characteristics (e.g., public or private programs, length of preschool days, organizational supports for physical activity). Process evaluation methods included both direct observations (OSRAC-P [31]) and ratings by trained researchers of physical activity opportunities provided by teachers and children’s physical activity during those opportunities. Additionally, research personnel used and investigator developed checklists (classroom rating scales), teachers’ self-reports of intervention completeness, fidelity measures, barriers to implementation and children’s responsiveness to the SHAPES intervention. Finally, we obtained preschool directors’ self-reports of practices related to physical activity with interviews and research personnel assessments of implementation and preschool environments using rating scales. Data from these multiple sources will be triangulated to assess fidelity and completeness of intervention implementation in a manner similar to previous work by members of the investigative team [32,33].

## Discussion

At the time this investigation was initiated, to our knowledge no studies testing the effects of a multi-component intervention targeted at increasing physical activity energy expenditure in children attending preschools were in the public health and educational literatures on physical activity. Many previous investigations have focused on school-age children and have employed single intervention strategies. Additionally, we applied a rigorous measurement protocol that used objective instrumentation to evaluate the effects of the intervention on physical activity and sedentary behavior. Further, and importantly, this study is one of few to extensively conduct process evaluation to provide thorough documentation of the SHAPES intervention implementation. A recent report from the U.S. Department of Health and Human Services noted that the preschool and childcare environments are promising settings for increasing physical activity in children, and this study addresses several recommendations from the report [34]. The effectiveness of those recommendations are yet to be determined in a manner that will allow researchers and practitioners to provide better evidence-informed policies and practices in preschool settings.

## Abbreviations

MVPA: Moderate-to-vigorous physical activity; PA: Physical activity; BMI: Body mass index.

## Competing interests

The authors declare that they have no competing interests.

## Authors’ contributions

KAP helped conceive of the study, procure the funding, direct data acquisition and drafted the manuscript. WHB and RPS helped conceive of the study, procure the funding, direct data acquisition and critically revised the manuscript. MD and CLA helped conceive of the study and its design, procure the funding, analyze the data and critically revised the manuscript. RRP is principal investigator and conceived of the study and its design, procured the funding, directed data acquisition and critically revised the manuscript. All authors read and approved the final manuscript.

## Pre-publication history

The pre-publication history for this paper can be accessed here:

http://www.biomedcentral.com/1471-2458/13/728/prepub
